# LoRaWAN Mesh Networks: A Review and Classification of Multihop Communication

**DOI:** 10.3390/s20154273

**Published:** 2020-07-31

**Authors:** Jeferson Rodrigues Cotrim, João Henrique Kleinschmidt

**Affiliations:** Center of Engineering, Modeling, and Applied Social Sciences, Federal University of the ABC, Santo André 09210-580, Brazil; joao.kleinschmidt@ufabc.edu.br

**Keywords:** internet of things, LPWAN, LoRaWAN, multihop, mesh networks

## Abstract

The growth of the Internet of Things (IoT) led to the deployment of many applications that use wireless networks, like smart cities and smart agriculture. Low Power Wide Area Networks (LPWANs) meet many requirements of IoT, such as energy efficiency, low cost, large coverage area, and large-scale deployment. Long Range Wide Area Network (LoRaWAN) networks are one of the most studied and implemented LPWAN technologies, due to the facility to build private networks with an open standard. Typical LoRaWAN networks are single-hop in a star topology, composed of end-devices that transmit data directly to gateways. Recently, several studies proposed multihop LoRaWAN networks, thus forming wireless mesh networks. This article provides a review of the state-of-the-art multihop proposals for LoRaWAN. In addition, we carried out a comparative analysis and classification, considering technical characteristics, intermediate devices function, and network topologies. This paper also discusses open issues and future directions to realize the full potential of multihop networking. We hope to encourage other researchers to work on improving the performance of LoRaWAN mesh networks, with more theoretical and simulation analysis, as well as practical deployments.

## 1. Introduction

The Internet of Things (IoT) aims to enable heterogeneous devices to communicate and cooperate to provide smart services in different environments transparently to the user. In the next few years, billions of IoT devices will be deployed around the world, enabling smart systems for different applications [1]. Such applications include smart cities, smart farming, health care, manufacturing, transportation, and many others [2]. Wireless networks are essential for these applications to cover a wide area in a city, building, or a farm [3]. Typical wireless technologies used for this goal, such as ZigBee, Bluetooth, and Wi-Fi, have a range of few meters or tens of meters [4]. They can use multihop communication in mesh network topologies to expand the coverage area [5]. In recent years, the Low Power Wide Area Networks (LPWAN) were developed to provide a feasible solution for applications that require a wide area coverage and energy efficiency [6]. The most prominent technologies for LPWAN are Long Range Wide Area Network (LoRaWAN) and SigFox in the unlicensed bands and Long Term Evolution for M2M (LTEM) and Narrowband IoT (NB-IoT) [7] in the licensed bands. Long Range (LoRa) is one the most used in applications because of the facility to develop private networks operating in the unlicensed frequency bands (868 MHz in Europe and 915 MHz in USA and Brazil) [8,9,10,11]. LoRaWAN technology promises long-range communication with low data rates and low energy consumption. Some tests have shown that the technology may achieve several kilometers in rural environments or open areas. However, in scenarios with obstacles or inside buildings, the range achieved by LoRaWAN is significantly less due to attenuation and fading effects, resulting in packet losses and errors [11]. In urban areas, a lot of obstacles may degrade the signal, thus also decreasing the coverage area. Rural areas are very dependent on the terrain topology, where a mountain could create a shadow area, for example. A device in an adverse condition will require more power to transmit, and by consequence, the energy consumption will increase and the lifetime of the device will decrease. The LoRaWAN network is composed of end-devices that transmit data to gateways, forming a star network topology. The specification allows only one hop between end-devices and the gateway [8,9,10]. Multihop networks are well known for extending coverage and improving the energy efficiency of wireless networks, extending battery life due to lower transmission power when compared to single-hop networks. Some open research issues in LoRaWAN networks are the scalability and the network capacity. Several works have shown that dense networks with many devices may degrade the overall network performance with longer delays and low reliability [12,13,14]. LoRaWAN uses ALOHA as the Medium Access Control (MAC) protocol, which does not perform well when traffic load or node density increases, due to interference and packet collisions [15,16]. Multihop strategies may also contribute to improve scalability, capacity, and reliability, as pointed in [12,15].

Many authors have proposed multihop solutions for LoRaWAN [17,18,19,20,21,22,23,24,25,26,27,28,29,30,31,32,33,34,35,36,37,38,39,40], where some devices act as relays to other devices. The forwarding mechanism is a crucial choice for a multihop LoRaWAN, as it may affect the performance of the network, in terms of throughput, reliability, latency, and energy consumption. Some works propose mechanisms using intermediate nodes as a simple relay using only LoRa physical layer, while others propose routing protocols, forming more complex mesh networks. Another important choice in LoRaWAN is which node will be the intermediate node: the end-device or the gateway. In this paper, we provide a comprehensive review of multihop LoRaWAN networks, analyzing and classifying the existing proposals. With this classification, we give a better understanding of the current situation of multihop LoRaWAN, identifying the most promising approaches, and providing research challenges and future directions. The paper is organized as follows. Section 2 introduces the LoRaWAN main concepts, and Section 3 presents related work on multihop LoRaWAN networks. In Section 4, we present a classification of the devices and features to be used in multihop LoRaWAN, while Section 5 presents network topologies and applications. In Section 6, we discuss open issues and future research. Finally, Section 7 gives the final considerations.

## 2. LoRaWAN

LoRaWAN is a data link layer protocol developed by LoRa Alliance in 2015 to provide a low power connectivity solution to battery-powered devices [8,41]. The current LoRaWAN specification is 1.1 [9], and the most used specification is 1.0.3 [10]. The physical layer used by LoRaWAN is called LoRa [42], developed by Semtech company and based on the Chirp Spread Spectrum (CSS) modulation [43]. The CSS modulation uses a Spreading Factor (SF) to spread the information over the frequency. The SFs are orthogonal to each other and define the number of chirps per signal symbol. There are six possible SFs in LoRa networks, from 7 to 12, that determine the number of bits necessary to transmit the same amount of data. A higher number of bits per symbol increases the capability of the receiver to demodulate the message. Higher SF means that more bits are necessary to send the same information. However, it is possible to deploy the end-device further from the gateway. The orthogonality between SFs guarantees that different end-devices could transmit their packets using the same frequency but using different SFs. There are three available bandwidths: 125, 250, and 500 kHz. Usually, networks use a 125 kHz bandwidth. The relation between SF, bandwidth, and packet size is essential to determine the Time on Air (ToA), which is the total time a single transmission uses the air interface to send a packet. Two packets with the same size, using the same bandwidth but with different SF, have completely different ToA. For comparison, to transmit a packet with 50 bytes using 125 kHz bandwidth, the ToA is 0.113 s for SF 7, while using SF 12 the ToA is 2.62 s. Considering only the ToA, the latency to deliver a packet is at least 20 times higher with SF 12 than SF 7. A more detailed comparison, including other SFs, is found in [13]. The SF, bandwidth, and packet size chosen directly affect the throughput of the link [44]. A device transmitting with a low value of SF has a higher throughput than a device transmitting with a high value of SF. However, the increase in SF also increases the range of the transmission [45]. Table 1 summarizes the relation between the SF, throughput, and ToA considering a packet with different payload sizes, 125 kHz bandwidth, and without the duty cycle restrictions.

**Figure 1:**
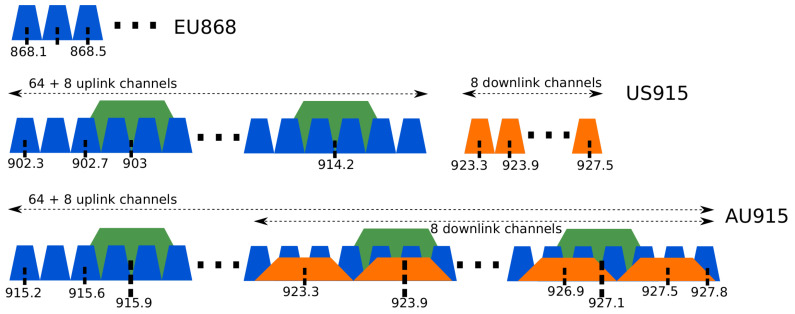
Long Range Wide Area Network (LoRaWAN) channels for Europe, USA, and Australia.

LoRa uses Industrial, Scientific, and Medical (ISM) frequencies, and every country or region has its frequency band. Europe uses 868 MHz, while the USA, Brazil, and Australia use 915 MHz, for example. Each country also uses a sub-band frequency scheme to create channels of transmissions. Each sub-band is composed of a number of frequencies called channels. Australia, for example, uses sub-bands composed of eight channels (frequencies) using 125 kHz bandwidth. The sub-band is essential to separate networks in the same area by using different frequencies. Figure 1 presents the channel scheme for Europe (EU868), United States (US915), and Australia (AU915) [46]. The European regulation specifies that the network channels can be freely attributed. However, all end-devices shall implement the three default channels (868.1 MHz, 868.3 MHz, and 868.5 MHz). The United States and Australia have a similar channel scheme with 64 uplink channels utilizing 125 kHz bandwidth, and incrementing linearly by 200 kHz. Both regions also implement eight channels with 500 kHz bandwidth and increment linearly by 1.6 MHz. The difference between US915 and AU915 is the downlink channel. Both have eight channels utilizing 500 kHz bandwidth, but US915 uses a separated band from the uplink scheme, while in AU915 the downlink overlaps the uplink frequencies. Countries and regions may also determine the amount of time one single device could use the channel by implementing duty cycle restrictions. In Europe, the duty cycle is 1% [47].

**Figure 2:**
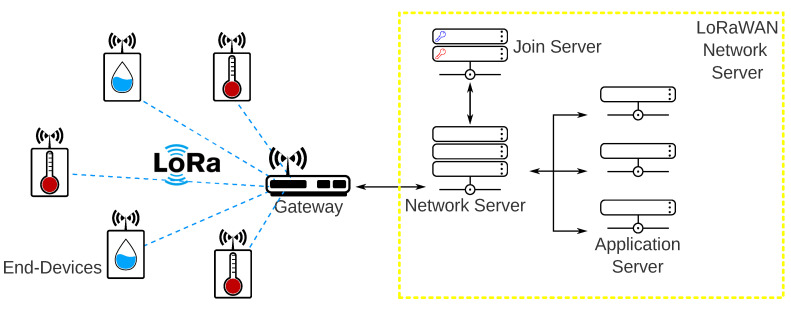
Typical LoRaWAN network.

Typically a LoRaWAN network is a star-of-stars topology composed by one or more gateways, end-devices, and a LoRaWAN Network Server. Figure 2 presents the basic topology of a LoRaWAN network. The end-devices are the nodes responsible for collecting data or actuating and generating LoRaWAN packets. The gateway is responsible for receiving the LoRaWAN packets and forwarding them to the LoRaWAN Network Server through an IP based network. It only handles in the physical layer, being unable to decode the packets and know their content. The LoRaWAN Network Server is a short way to describe a more sophisticated element composed of three main components: a Network Server, an Application Server, and the Join Server. The main functions of the LoRaWAN Network Server are as follows.

- Network Server: It is the central element of a LoRaWAN network responsible for managing the MAC layer. The Network Server is responsible for many management functions in the network, such as the verification of the end-devices addresses, packet acknowledgment, frame count, and answers to end-devices requests. Furthermore, the Network Server forwards the messages to the Application Server and Join Server, and manages the downlink messages queue.
- Application Server: The Application Server is responsible for forwarding all received packets from the Network Server to the specific associated application. In the same way, a message from one application is forwarded by the Application Server to the Network Server.
- Join Server: The Join Server takes care of the end-devices authentication process, generating and distributing the authentication keys. There are two authentication methods allowed on a LoRaWAN network: the Activation by Personalization (ABP) and the Over-The-Air Activation (OTAA). Section 2.1 describes both authentication methods in detail.

**Figure 3:**
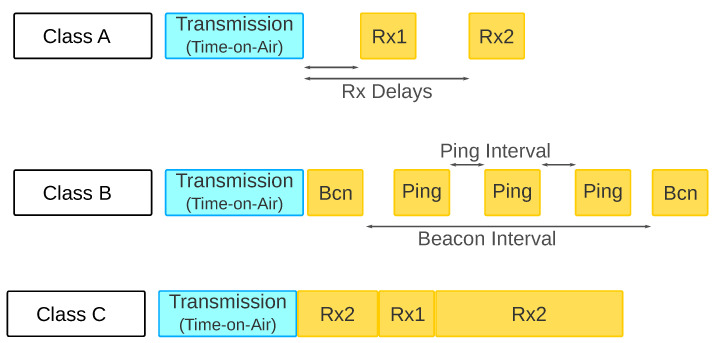
LoRaWAN classes.

The end-devices transmit their data to one or more gateways, and the gateways forward the message to the Network Server. No communication between end-devices is allowed in the default standardization. The end-devices follow one of the three possible classes of operation, as shown in Figure 3.

- Class A: In this class, end-devices can send packets at any time they want. After each transmission, the end-device must open one or two reception windows. If the end-device receives a packet in the first reception window, the second one will be kept close. All end-devices must join a network in Class A mode and, according to the Network Server request, can change their behavior to another operation class. The ALOHA protocol controls the medium access since it is suitable for energy-constrained applications. However, the ALOHA protocol is well known to provide low network throughput in dense networks because of a high number of packet collisions [15,16]. The ALOHA limitation in LoRaWAN was already discussed in terms of scalability and reliability in several works [12,48].
- Class B: Defines a mechanism that allows end-devices to open more reception windows than the default ones. The end-device will open periodically new reception windows following the Network Server demands. The gateway and end-devices use a beacon message to get synchronism between them. The new reception windows receive the ping name.
- Class C: Opens the two reception windows and keeps the second one open until the next uplink transmission. Class C must be used only by non-energy constrained devices.

LoRaWAN end-devices implement a mechanism called Adaptive Data Rate (ADR) to improve the use of network resources. Based on data collected from the end-device, the Network Server could request this end-device to change its characteristics, such as the SF, the bandwidth, or the transmission power. Each country may specify a different Data Rate (DR) [49], which is a number related to a specific bandwidth and SF, used to inform the end-devices of the new characteristics of the ADR process. Each end-device decides who is responsible for managing the ADR: the end-device itself or the Network Server.

### 2.1. LoRaWAN Security

Information security is an essential requirement for all IoT levels, and the LoRaWAN protocol was developed to provide a secure connection to all the devices. Furthermore, the security solution to the LoRaWAN networks also concern about energy consumption, low levels of complexity, low cost, and high scalability [50]. LoRaWAN provides end-to-end security, from the end-device to the application, using the standard AES cryptographic algorithm.

Every end-device has a unique identifier called *DevEUI*, an application identifier called *AppEUI*, and a application key called *AppKey*. The *AppKey* is used to generate the Network Session Key (*NwkSKey*) and the Application Session Key (*AppSkey*). The *NwkSKey* is used to provide integrity to the data and MAC commands. The *AppSKey* provides end-to-end cryptography. The LoRaWAN implements two different network join methods handled by the Join Server to authenticate an end-device [51].

The first authentication method is called ABP, which specifies that all the information necessary to authenticate a device is previously set up in the end-device and the Join Server. The ABP mode excludes the need of the end-device to exchange authentication messages with the Join Server. The network manager is responsible for managing the join procedure.

**Figure 4:**
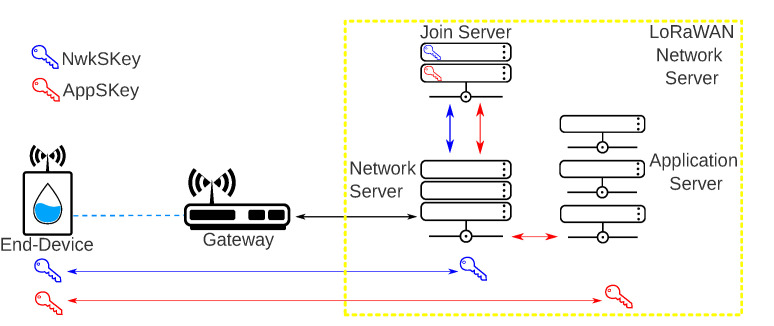
LoRaWAN security architecture.

The second method is called OTAA and requires a handshake between the end-device and the Join Server. Only end-devices can initiate a join procedure, and they do it every time a new device wants to join a network or a device loses connection with the Network Server. The end-device starts the join procedure sending a join request message to the Join Server that will accept or not the entrance of the end-device on the network. The Join Server sends back a join accept if the end-device is authorized to join the network. When the Join Server receives the join request, it generates the *NwkSKey* and stores it in the Network Server. The Join Server also creates the *AppSKey* and stores it in the Application Server. Both keys (NwkSKey and AppSKey) are sent to the device with the join accept. Figure 4 presents the LoRaWAN security architecture with the respective authentication keys.

## 3. Related Work

This section presents an overview of the existing works in multihop for LoRaWAN. In general, all approaches consider intermediate nodes (end-devices or gateways) to expand the coverage of the network. A packet transmitted by an end-device will be forwarded by the intermediate nodes until it reaches a gateway connected to a Network Server. These intermediate devices may perform simple relay functions or complex routing protocols.

Table 2 presents a summary of existing works on multihop LoRaWAN, showing the protocol level used (LoRa or LoRaWAN); the scenario used for testing; and if the work is a theoretical proposal (T), a simulation (S), or a practical testbed (P). It can be seen that some works consider only the LoRa physical layer, while others use LoRaWAN characteristics. Most of the previous work are practical implementations of the aforementioned proposals.

### 3.1. Routing Proposals

Several works that introduce multihop networking propose the use of a routing protocol, either based on existing protocols for ad hoc and mesh networks, or even new solutions. The “LoRa for the Internet of Things” article was the first work proposing a multihop LoRa-based network in 2016 [17]. The authors proposed a new protocol called LoRaBlink that works over the LoRa physical layer and aims to cover several points not specified by LoRAWAN v1.0, including multihop communication. The protocol integrates MAC and routing at the same layer in a synchronous flooding based protocol. The work evaluated the protocol in a university campus, in a testbed composed of six nodes and one sink deployed in different places. The results have shown that 80% of the packets were reliably delivered.

In [18], the authors proposed the use of Concurrent Transmission Protocol (CT), a novel flooding routing protocol successfully used in IEEE 802.15.4 networks. The CT protocol does not demand a routing table, and the flooding mechanism does the synchronization of the nodes. The results for the indoor proposed scenario showed that the protocol enhances the coverage of LoRa and achieves a reliable packet delivery rate. The authors do not present an energy consumption analysis of the protocol.

The work presented in [19] combines the Hybrid Wireless Mesh Protocol (HWMP) and Ad hoc On-Demand Distance Vector (AODV) protocol to create a lightweight protocol for LoRa. The proposed solution works with the deployment of intermediate nano-gateways (which do not implement full LoRaWAN), and the routing mechanism is transparent to end-devices and the network server. The protocol is evaluated only in terms of route construction delays.

The authors of [20] proposed an energy-efficient multihop communication solution (e2McH) where routes are constructed based on energy consumption, residual battery life, and traffic rate. Simulations results show an improvement of 15% in energy consumption compared to the single-hop lorawan.

In [21], the authors proposed a linear multihop communication over LoRa to monitor the ancient underground water distribution systems in Siena, Italy. The authors used a simple routing protocol and a synchronization mechanism, where the end-devices use a wake-up time transmission scheme to minimize energy consumption. The results showed that the proposed solution is reliable, and the synchronization mechanism reduces the energy consumption by 50% when comparing to non-optimal wake-up time. A linear topology may be the only viable option in some applications, such as mines and pipelines. In [22], the authors also considered a linear multihop network based on lora. They proposed a simple routing protocol to enhance the coverage area. The authors also added a synchronization mechanism to the end-devices and implemented the protocol in a real environment. The performance was analyzed using the throughput and reliability of the testbed, without power consumption analysis.

The work in [23] proposed a routing protocol over a LoRa physical layer based on a child-list to create routes. It uses a polling-based approach, where the devices listen to requests of the gateway. Battery-powered end-devices are used as intermediate nodes. The solution was tested at the university campus and in an indoor building. The results showed the increase of packet delivery rate using the multihop approach compared to direct transmission. They also showed that the number of nodes in the network that could transmit simultaneously decreases compared to the star topology.

The authors of [24] adapted the Destination-Sequenced Distance Vector (DSDV) routing protocol to work in LoRaWAN. The proposed solution classifies the nodes into two types: the routing nodes and the leaf ones, where the routing devices are non-energy constrained. The authors tested the solution in linear and bottleneck scenarios in a prototype system. They conclude the solution is feasible even for the duty cycle limitations.

The research in [25] proposed a full protocol solution over LoRa physical layer, including TDMA access, collision avoidance, synchronization, and routing mechanism to provide a low latency solution. The authors deployed end-devices on a university campus to do the tests. The results showed that the protocol has high data reliability and low latency, but energy consumption was not analyzed. They concluded that the proposed solution is suitable for agriculture applications and in tunnels.

The authors of [26] presented a networking clustering based on the spreading factor to improve the performance of multihop LoRa networks. They proposed a tree-based SF clustering algorithm (TSCA), which assigns the nodes to several subnets. The strategy explored the possibility of parallel transmission in LoRa using multiple SFs. The authors analyzed the solution with simulation and practical scenarios, and they concluded that an efficient multihop LoRaWAN network needs to consider the SF allocation.

The work in [27] proposed a new architecture to LoRaWAN to solve the problem of monitoring underground infrastructure. The architecture is composed of sensor nodes forming a LoRa mesh network sending data to a repeater node, which acts as a sink for the sensors. The repeater node communicates directly with the main gateway, and it may be a simple battery-powered end-device not connected to the Internet. While the sensor nodes act as routers in the mesh network, the repeater node acts as a relay that forwards the messages to the gateway. The authors conducted two field tests as a proof-of-concept. Both repeater and router nodes are energy-constrained, single-channel (repeater with two radios), and implement a synchronization mechanism. The results showed the improvement in the packet delivery ratio for underground devices, but no energy consumption analysis was presented.

The first proposal of routing protocol in a LoRa network, using a typical IP stack, was presented in [28]. The authors proposed a new MAC layer to deal with IPv6 Routing Protocol for Low Power and Lossy Network (RPL) routing protocol, and conclude that enabling RPL over LoRa is an open issue and needs to be better tested. In [29], the authors propose to use the Time Slotted Channel Hopping (TSCH) protocol over LoRa physical layer to provides a multihop IPv6-based solution. The experiments testbed include real devices, and the authors demonstrated the possibility to implement an IPv6 network over LoRa.

In [30], the authors state that many routing proposals for LoRaWAN do not consider constraints such as packet size, duty cycle, and time for downlink transmission. They proposed a new class G to the LoRaWAN standards, where they include a gateway-to-gateway communication using a routing protocol based on AODV. The authors used a simulation scenario to validate the proposal and concluded the communication gateway-to-gateway had a reasonably good performance in terms of throughput and delay.

### 3.2. Relay Proposals

The intermediate nodes used to form multihop networks may perform simple tasks comparing to routing, that is, acting only as repeaters. We call these nodes as relay nodes, that retransmit the packets received to the next node, without the need of establishing routes. Although simpler than routing, this approach may be attractive for LoRaWAN, since routing protocols may include unnecessary complexity, thus consuming more resources of the constrained devices. Relay nodes may also act as a sink for a mesh network, as presented in [27] and discussed in the previous subsection.

Only in 2018, the first papers started to present solutions using relay nodes. In [31], the authors proposed a new device to the LoRaWAN networks called e-Node, which is a relay node developed using a multichannel and non-energy constrained gateway. The main idea is to increase the coverage area with a transparent node to the network. In [32], the same authors used an industrial scenario to validate and implement a proof-of-concept prototype of the e-Node, but using an end-device as an intermediate node. They measured several messages delays and concluded the proposed solution could improve the coverage of the network with high reliability of packets sent by distant end-devices.

The work in [33] proposed to use a simple relay device to increase the LoRaWAN coverage area for rural areas. The authors suggested deploying the relay nodes knowing the places that are not covered by the gateway. The authors proposed a simple message forwarder and a synchronization mechanism. They showed that energy consumption decreases with the addition of the relay node to deliver the packets. They also notice an increase in the coverage area and reliability.

In [34], the authors created a hybrid intermediate device capable of executing the ABP join procedure of remote nodes, while being validated by the network server using OTAA. This mechanism avoids the remote nodes to do the OTAA and decreases the number of messages on the network. However, the authors do not discuss any mechanism to forward the data packets. In the results, they showed the packet delivery rate achieves 100% in the proposed scenarios.

The research in [35] proposed a multihop technique based on three main mechanisms: opportunistic forwarding, opportunistic transmission, and networking coding. The relay node will only transmit the packets of end-devices that send their packets to the gateway without success. The simulation results presented a similar packet delivery rate compared to directed transmission or simple multihop. However, the proposed mechanism requires fewer transmissions.

The work proposed in [36] uses an Automatic Repeat Request (ARQ) mechanism and relay nodes to improve reliability on LoRa networks. The relay nodes chose the best parent to transmit the data based on collected RSSI. The authors implemented a real testbed on a university campus and presented results only for packet reliability.

In [37], the authors analyzed two architectures for multihop LoRaWAN in smart cities. In the first architecture, an end-device functions as a relay node to extend the coverage area. In the second architecture, called star-of-stars, a group of remote devices forms a cluster sending data to a cluster gateway, which acts as a relay sending the information to a central gateway. The authors implemented a prototype of both architectures, but did not present any new forward mechanism. The results showed a decrease in energy consumption for two or three hops compared to single-hop communication. Moreover, this work discusses some challenges and future work for multihop LoRaWAN networks.

### 3.3. Analyses and Other Approaches

Other works studied the issue of multihop for LoRaWAN and LPWANs. Some of them presented theoretical approaches with analytical models and simulations [38,39,40], simple solutions [52,53,54,55,56], or generic proposals for LPWANs [57,58] that may be extended to LoRaWAN. Another interesting approach is presented in [59], where the authors proposed a control plane and data plane separation for multihop networks. The main idea is to use LoRaWAN for the control plane and another multihop technology to the data plane, like ZigBee. An implemented testbed showed the feasibility of the proposed approach. The works presented in [52,53,54,55,56] discuss some practical issues about multihop LoRaWAN, but the authors give few details of the implementations, so they are not discussed here.

In [38], the authors analyzed the energy efficiency of the star and mesh topology. They also proposed an analysis to find the best relations between spreading factor, transmission power, distance, and bandwidth. In terms of energy consumption, they concluded that the best choice between direct or multihop transmission depends on the end-to-end distance between the sender and the gateway. In [39], the authors presented a model to analyze the energy consumption of single-hop and multihop LoRaWAN networks. The authors consider a network formed by several rings around the gateway in the multihop approach. The simulations used MatLab, and the authors concluded the nodes near the gateway consume more energy than the nodes far from it in the multihop scenario. On the other hand, for the single-hop scenario, nodes near the gateway present a better energy efficiency. Another theoretical work was presented in [40] for different possible multihop set-ups for LoRa networks with three hops (or layers) to the gateway. The results demonstrated that some topologies could improve packet delivery ratio and energy consumption when compared to traditional single-hop LoRaWAN.

In [57], the authors consider a generic LPWAN based on the TDMA MAC layer. They proposed a Distance-Ring Exponential Stations Generator (DRESG) framework, which evaluates the performance and establishes optimal routing connections for multihop communications in the uplink. Their results showed that multihop might improve network lifetime and balance the energy consumption among all nodes in the network. In [58], the same authors proposed a protocol stack for LPWANs called HARE, which permits single-hop and multihop. It is composed of several techniques in different communication layers, including network synchronization, TDMA channel access, adaptive transmission power level, network association process, and energy-aware routing protocol. The protocol was implemented in real hardware platforms and showed high reliability and low energy consumption.

## 4. Classification of LoRaWAN Devices for Multihop

In this section, we present and define a classification for devices in a LoRaWAN mesh network based on the function the devices have in the network. The main idea of the proposal for multihop networks is to keep the default LoRaWAN standardized device types and to define a new function to the end-devices and gateways. The end-device is the one capable of collecting data or acting in the network and can generate LoRaWAN packets. The gateway provides a connection between end-devices and the Network Server, and never generates a new LoRaWAN packet. We admit a gateway is a device with the capability to change networks or split networks.

A new classification based on functions performed by devices is necessary to describe and separate the regular devices from the new intermediate devices on a multihop network. The intermediate device could act as one of the two new functions proposed: a relay or a router. We define the gateway connected to the Network Server as the main gateway, and the other possible gateways as the intermediate gateways. All main gateways are connected to the Network Server and, by definition, they change from LoRaWAN Network to IP Network. One network could have more than one Main Gateway. All gateways could have the capability to connect to the Internet, but to work as an intermediate gateway, the Internet connection must be down. An intermediate gateway could change its status to be a main gateway if necessary. A network manager may be responsible for making this decision based on the transmission quality. A gateway is set as an intermediate device if the connection between gateways is more reliable than to use a 4G or 5G connection, for example. Remote areas are suitable scenarios to use intermediate gateways to improve coverage.

The relay is a device capable of forwarding all received messages from other end-devices. The relay could work in the physical layer only (LoRa) or the data link layer (LoRaWAN). The intermediate devices with relay function could work as a regular end-device at the same time. The relay device keeps the LoRaWAN network characteristic forbidding communication between end-devices.

The router devices, as the name suggests, require the implementation of a routing protocol. The routing protocol requires, at least, the data link layer, and some works insert a network layer over the LoRaWAN [28,29]. In a routing network, all the router devices may also act as a regular end-device. The routing protocol adds the possibility of communication between end-devices.

### 4.1. Device Characteristics

The devices performing relay and router functions share three main characteristics as follows.

- Radio: The intermediate nodes could have a single or multichannel hardware radio. If the devices use a single channel, there will be severe limitations. The intermediate device will have to receive packets in only one frequency and DR at the same time. By default, all end-devices can transmit in eight different frequencies in a pseudo-random frequency hopping technique. If the intermediate device is multichannel, it will be able to receive up to eight packets at the same time with different frequencies or SFs. However, multichannel radio hardware requires a non-energy constrained device to work properly.
- Energy Constraints: The energy capabilities of the intermediate device are important to determine how many features the device could have. Generally, a constrained device is not adequate to perform complex activities because the energy consumption will be high and may decrease the device’s lifetime. Here, we consider a multichannel device as a non-energy constrained object because the radio needs to consume more energy, and even the development boards require at least a Raspberry Pi to support the radio. Furthermore, a gateway is always a non-energy constrained hardware.
- Smart: The smart characteristic refers to the capability a device has to perform any high-level feature. A non-smart device will only be responsible for receiving and forwarding a packet, that is, a very simple relay. A smart intermediate device will be able to execute other features, some of them are described below.
  - *Routing:* We classify any routing technique as a smart feature because it requires the devices to make decisions to create or select routes. Only router devices may have this function.
  - *White-List:* To minimize the number of packets that an intermediate device handle, it is possible to implement a white-list where the device only forwards the packets from the devices on the list, thus decreasing the total amount of messages in the network. The white-list may also be useful to avoid routing loops.
  - *Loop avoidance:* Using the header of the LoRaWAN packet, a device may add information if the packet is new or a forwarded one. A packet tagged as forwarded when received again by a relay could be dropped to avoid loops.
  - *Synchronization:* The synchronism mechanism is essential to the intermediate device to know when it will receive a packet and, by doing this, save energy.
  - *Buffering:* The total amount of data received by the intermediate device could exceed the transmission capacity of the device, or the duty cycle restriction. The intermediate device could manage a buffer and a queue to deliver the received packets and minimize the losses.

### 4.2. Devices Classification

The router and relay functions can be implemented in an end-device or gateway device, so we admit a new nomenclature to simplify the understanding. The end-device with relay function will be called Relay-Device, and when working as a router will be called Router-Device. The gateways follow the same rule, so there will be a Relay-Gateway and a Router-Gateway.

The Relay-Device is a simple message forwarder and could work directly on the LoRa physical layer or LoRaWAN link layer. The Relay-Device could work without any smart feature, but to improve the efficiency of the solution, a synchronization mechanism can be implemented, for example. A Relay-Device always sends the packets to another relay or gateway, and always participates in only one network. The hardware requirements include single- or multichannel radio, and the hardware definition will affect the energy requirements. A multichannel radio needs a non-energy constrained device to be reliable.

The Router-Device implements a routing protocol to create a route between the end-device and the gateways (intermediate or main gateway). From the related work presented in Section 3, we can notice that there are several possibilities for a routing protocol, from a simple flooding mechanism to one that is more sophisticated, including the IPv6 stack. The routing protocol is a smart feature, so all Router-Devices are smart objects. A single or multichannel radio supports the implementation of a Router-Device, and this decision will affect the energy requirements. A multichannel device requires a non-energy constrained hardware.

The Relay-Gateway and Router-Gateway are always developed using multichannel radio, so they are non-energy constrained devices. The difference between them is that the Relay-Gateway could be a non-smart object. Besides the similarities with intermediate end-devices, a Relay-Gateway or Router-Gateway never collects data or act in the network, and never generate a new LoRaWAN packet. The gateway solution could split the network by choosing different sub-band frequencies for reception and transmission. A Router-Gateway can function as a border gateway, participating in the end-devices subnetwork and the gateways subnetwork at the same time.

Table 3 summarizes the classification of the devices, presenting the nomenclature, characteristics, and the most important details of each one.

Table 4 presents a classification of the related work in terms of the types of devices used as intermediate nodes and their characteristics. The theoretical works and the other approaches of Section 3.3 are not classified in the table. From the table, it is possible to observe that most works proposed a solution using an end-device with single-channel radio. Moreover, most works use non-energy constrained devices with smart capabilities. The works are balanced in terms of using relays or router as a solution to multihop LoRaWAN.

### 4.3. Micro-Server Gateway Solution

An essential characteristic of the intermediate gateways is the hardware capabilities, as the multichannel radio requires a robust platform to support it, like at least a Raspberry Pi. This availability of robust hardware allows the development of new and high-level features. One possible solution that could be implemented in the intermediate gateways is to extend Network Server functions. One possible application is to create a micro-server to handle functions such as the join procedures and ADR. A secure LoRaWAN network requires the OTAA join procedure, and the number of hops in a multihop scenario could be a problem. The join procedure will increase the total amount of messages in the network, and if the duty cycle needs to be respected, the overall time could increase. As explained in Section 2, ADR is a mechanism that could be controlled by the end-device. However, for better results, the best approach is to leave all the decisions to the Network Server. Using intermediate devices, the downlink packets to inform the end-device the new transmission setups could be lost or take a long time to be delivered due to duty cycle restrictions, and the total amount of data in the air interface will increase. The micro-server solution could implement the ADR mechanism to control a list of end-devices connected to it. No work in the literature has studied these characteristics so far.

## 5. Network Topology

**Figure 5:**
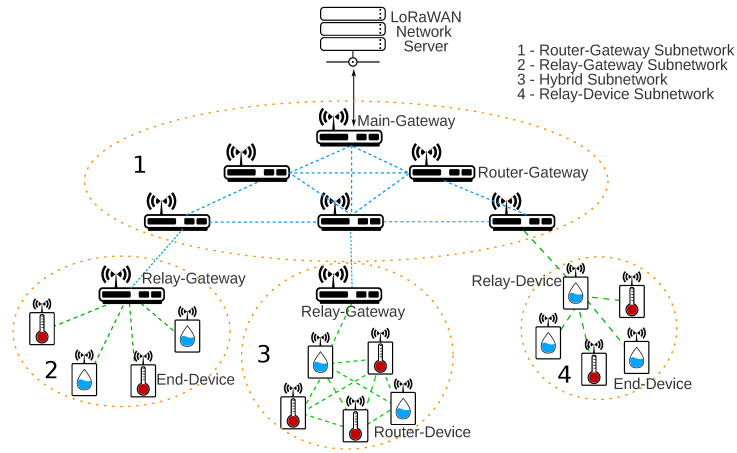
LoRaWAN mesh network.

The insertion of an intermediate device changes the LoRaWAN network topology from star-of-stars to several new possibilities. For a better understanding, it is important to contextualize the relay and router nodes in a mesh network topology. Figure 5 presents a big picture of the multiple mesh LoRaWAN network possibilities based on the concept of mesh networks presented in [60]. The subnetwork *1* represents the communication between gateways using a routing protocol. The Main Gateway is connected to the Network Server, and the others are Router-Gateways. The subnetwork *2* presents a Relay-Gateway providing connection to the end-devices. In the subnetwork *4*, the relay function is performed by an end-device, which could also act as a regular end-device collecting data. The subnetwork *3* presents a solution with router-devices. In subnetworks *2* and *3*, there is a Relay-Gateway connecting the remote devices with a gateway network level (subnetwork *1*). The next subsections present and describe the possible new LoRaWAN multihop topologies.

### 5.1. Relay Topology

**Figure 6:**
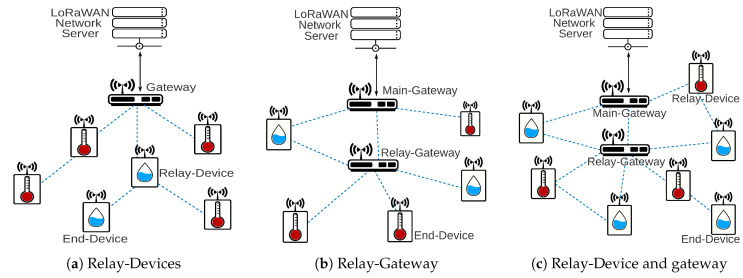
Architecture with relay nodes.

To provide or improve the connection of remote nodes it is possible to use relay devices. Figure 6a presents a solution using Relay-Devices, where the Relay-Device also works as a regular end-device. The solution is suitable for a smart building or industrial application where the end-devices have access to a power supply, for example. Figure 6b shows the relay solution with Relay-Gateways. One important difference between the Relay-Device solution compared to the Relay-Gateway is that the Relay-Device could be battery supplied or not, while the Relay-Gateway must be non-energy constrained. The Relay-Gateway uses a multichannel radio allowing the device to listen in at least eight frequencies, which permits the Relay-Gateway to concentrate the traffic from remote end-devices without any mechanism to select the frequency used. A rural scenario is very suitable to use a Relay-Gateway to connect remote devices to the main gateway.

Figure 6c presents an architecture composed of Relay-Devices and Relay-Gateways. An example scenario of a mixed relay network is a smart city where there is the need to connect remote devices and concentrate traffic at different points of the network. A Relay-Device could provide a connection to a remote end-device, and the Relay-Gateway could aggregate the data from a set of end-devices (and Relay-Devices) to deliver their messages to the main gateway.

### 5.2. Router Topology

**Figure 7:**
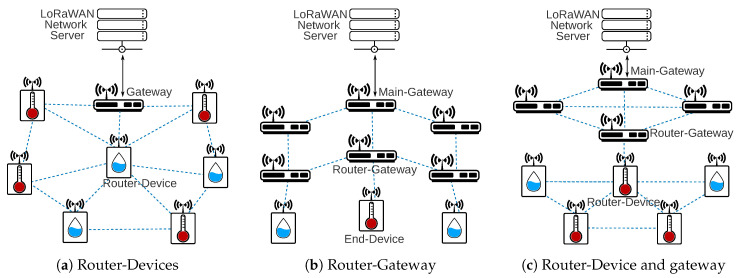
Architecture with router nodes.

The router topology describes the network architectures based on devices with routing capabilities. The routing protocols can work at the end-devices or at gateway level. Figure 7a,b depicts both solutions individually and Figure 7c puts in the same network infrastructure the two routing levels.

Figure 7a may represent a scenario like a smart building with a large number of nodes in a complex network with access to the power supply and with a lot of transmission barriers. As the end-devices have a lower cost compared to gateway hardware, it is possible to admit the use of Router-Devices for the scenario.

Figure 7b presents the end-devices connected to only one Router-Gateway. However, it is possible to admit that the end-device could have a connection to more than one Router-Gateway at the same time. In this case, the Router-Gateways would have to manage the duplicated packets in the network to avoid unnecessary data traffic. A farm with some points of energy infrastructure, such as a central irrigation pivot, is very suitable in using a Router-Gateway solution. Each Router-Gateway can work as a packet concentrator of some end-devices and determine a better route to forward the information through the main gateway. It is also important to admit every Router-Gateway could have a connection to another network, like 4G or 5G, disabled until the network manager decides to enable it.

Figure 7c shows the interconnection between distinct routing levels, one for the Router-Devices and another to the Router-Gateways. The routing protocol, the network manager, and physical limitations will determine if one or more Router-Devices will have a connection to one or more Router-Gateways. The scenario is relevant to provide a reliable connection to remote or restricted areas like underground deployment with the addition of routing gateway level.

### 5.3. Hybrid Topology

A hybrid topology is also a possible implementation, with part of the network using routers and another part using relays. Both routing or relay level could be implemented in the end-devices layer or gateway layer. Several end-devices could transmit data packets to a Relay-Device, and the Relay-Device would forward all data to a Router-Gateway. Finally, the Router-Gateway would send all the packets until they reach the main gateway. Another option is a subnetwork formed by Router-Devices connected to one Relay-Gateway that will forward the messages to the main gateway. The work presented in [27] uses the idea of a hybrid topology with Router-Devices connected to one Relay-Gateway. The authors connected several Router-Devices to monitor underground streets for a smart city proposal. All the Router-Devices data are forwarded to one root node. The authors called the root node as a relay. However, by the description, we classified the device as a Relay-Gateway because it is a multichannel radio and splits the networks.

### 5.4. Summary of Topologies

Table 5 summarizes the proposed topologies with possible application scenarios of each topology. The table also relates the works presented in Section 3 with the proposed topologies. Most of relay and router solutions use the topology showed in Figure 6a and Figure 7a, respectively. Only [31,37] (the authors used two scenarios, one with Relay-Devices and another with Relay-Gateway) uses a Relay-Gateway solution as shown in Figure 6b, and [30] uses a Router-Gateway as showed in Figure 7b. None of the related work presents a network solution like the Figure 6c and Figure 7c that uses Relay-Devices and Relay-Gateways or Router-Devices and Router-Gateways, respectively.

## 6. Open Issues and Future Directions

In general, existing literature has shown that multihop and mesh topologies may extend the coverage of LoRaWAN networks and potentially improve energy efficiency and reliability in certain scenarios. However, many open issues remain as challenges for the widespread adoption of multihop in LoRaWAN. As this feature is not present in current specifications, the solutions so far have to be implemented by the LoRaWAN network developers. A standardization on LoRaWAN mesh networks would lead to more adoption of multihop characteristics. Other wireless IoT technologies (Wi-Fi, Bluetooth, and ZigBee) have a mesh standard, which facilitates the adoption of multihop by applications. Some proposals of future research for multihop LoRaWAN are listed below.

- Power consumption: The energy consumption is of utmost importance in battery-powered nodes. Several techniques may be employed to increase the battery lifetime of the end-devices, such as energy harvesting, detection and decoding weak signals, and transmission using backscatter signals [11]. Cognitive radio is another promising approach to be explored in LoRaWAN. Multihop communication may benefit from all these techniques to save energy in constrained devices. However, many of the recent multihop proposals do not deal with energy issues, like the ones presented in [18,23,24]. Although these proposals could be applied in applications with no energy constraints, many scenarios where it is unfeasible to have an external power source could not use such solutions if the energy consumption is high, thus depleting the node’s battery. More work has to be done on analysis of the power consumption of relay nodes and routing protocols, which add new messages in the network. Real-world measurements in practical implementations, as well as analytical and simulation tools, may help in this challenge.
- Scalability and multihop optimization: The scalability of single-hop LoRaWAN has been extensively studied, and it is one of the main challenges of the technology. When the traffic load or the node density increases, the network performance can be severely affected [12,13,15]. Some works propose to improve scalability at the MAC layer level of LPWAN, using techniques such as scheduled MAC protocols, station grouping algorithms, adaptive transmission mode, and adaptive power control [14,58], or even using concurrent multiband technologies [15]. Multihop communication may also improve the network scalability, but many works presented in this review only show results for networks with few nodes and low traffic. Therefore, more research is needed on how and when multihop transmission may overcome single-hop transmission. Many issues remain open, such as the number of devices per relay, the number of relays, the number of hops to the gateway, and the energy consumption, as already mentioned. The requirements may be different in urban scenarios, rural areas, and industrial applications.
- Densification and Coexistence with other LPWAN technologies: With many LoRAWAN networks deployed in urban scenarios, coexistence issues must be considered. The performance of isolated networks will not be the same in scenarios with other coexisting devices, such as intermediate nodes for multihop communication. Coordination mechanisms between gateways and intermediate devices from different operators must be necessary. This problem is even harder considering the different sub-GHz technologies that use the same wireless spectrum, such as SigFox, IEEE 802.15.4g, and IEEE 802.11ah, causing interference at a larger scale. As described in [14], some applications may require that different technologies form one LPWAN, switching from one wireless standard to the other and also creating multihop LPWANs with different LPWAN technologies. This integration would require new routing protocols, handover mechanisms, and a virtualized LPWAN interface.
- Synchronization, Queuing, and Duty Cycle: In LoRaWAN mesh networks, the intermediate nodes require tight synchronization between nodes in every hop to the gateway. LoRaWAN specification provides synchronization using beacons, but only for Class B devices in single-hop networks. Recent work proposed some methods for synchronization for multihop, such as using the same idea of beacons for multihop [17,24], beacon flooding [27], TSCH scheme [29], or using synchronization by flooding with concurrent transmission [18]. More research has to be done using different methods, MAC schemes, and performance evaluation. One problem that may appear is that the intermediate node can not immediately transmit the received packet, thus temporarily storing the data. The node must have enough memory for buffering, which is limited in many end-devices. Moreover, the delay for the data packet will increase, leading to longer delays that may prejudice the application. Moreover, with more transmissions by the relay nodes, the duty cycle may be affected, compromising the network capacity. Data aggregation techniques may alleviate the number of transmissions and is another topic of research.
- Optimal placement of relays: The position of the relay nodes (or routers) in the network has a direct impact on both range extension and energy consumption. This question is similar to the optimal placement of multiple gateways (in single-hop networks). The optimal placement of relays depends on many factors, such as hardware characteristics of the nodes and the application.
- Intelligent LoRaWAN networks: There are many configurable parameters in LoRaWAN, such as spreading factor, bandwidth, coding rate, and transmission power, resulting in hundreds of possible settings. All these parameters must be optimally chosen considering the constraints and objectives of the application, such as minimizing the energy consumption or latency or maximizing the throughput and packet arrival. With multihop communications, the optimal configuration is yet more difficult to achieve by mathematical and statistical models, as pointed in [37]. In such situations, machine learning and deep learning are good candidates to be explored in forming efficient LoRaWAN networks.
- Security: The introduction of multihop topologies poses new challenges to security, like denial of service attacks (DoS) and traditional attacks at the routing layer, such as black hole, gray hole, wormhole, Sybil, flooding, and so on. The relay or router nodes are especially vulnerable to these types of attacks, which may compromise many end-devices in the network. Intrusion detection techniques may be required to detect and mitigate such attacks. Besides that, the network must have security mechanisms for node authentication and key management in addition to the already defined in the LoRaWAN specification.
- Gateway-to-gateway communication: Among all solutions proposed in the literature so far, few of them [30,31,37] studied the gateway as an intermediate node. Many applications may require this type of communication, such as several gateways covering a rural area, where only one of them has a stable Internet connection. Another open issue is how to handle server functions in intermediate gateways, such as join procedure and ADR, as discussed in Section 4.3.
- IPv6 over LoRaWAN: One promising solution to be used for multihop LoRaWAN is IPv6 adaptation, thus connecting the LPWAN to the Internet. Simple solutions were investigated in the literature [29], but solutions using the Static Context Header Compression and Fragmentation (SCHC) [61], an ultralightweight IPv6 adaptation layer for LPWANs, are still to be investigated.

## 7. Conclusions

LoRaWAN is an LPWAN technology widely adopted to connect several devices with low power and long-range communication. It allows building a private network without other third-party infrastructure, like SigFox and NB-IoT. Several authors have proposed multihop mechanisms to extend coverage, which were discussed in this paper. These solutions propose intermediate nodes to forward messages from other end-devices. The intermediate nodes in each hop could be either an end-device or a gateway, performing simple relay functions or complex routing protocols. The devices in previous work may have different constraints and features, forming different mesh topologies. We classified the existing solutions according to their characteristics, identifying the challenges in deploying LoRaWAN multihop networks. We showed that different applications could use different network topologies and intermediate nodes, depending on the requirements on coverage, reliability, energy consumption, and so on. Further, we presented some open issues and possible directions for practical large-scale deployment of LoRaWAN mesh networks. We encourage further developments in multihop communication to improve coverage, scalability, capacity, reliability, and energy efficiency of LoRaWAN networks.

## Figures and Tables

**Table 1:** Relation between Spreading Factor (SF), throughput, and Time on Air (ToA).

	50 Bytes Payload		Max Payload Size		
**SF**	**ToA (s)**	**Throughput (bits/s)**	**Payload**	**Max ToA (s)**	**Throughput (bits/s)**
7	0.113	3543.1	242	0.394	4913.7
8	0.205	1948.2	242	0.697	2777.6
9	0.369	1082.1	115	0.677	1358.9
10	0.698	572.8	51	0.698	584.5
11	1.478	270.5	51	1.479	275.9
12	2.629	152.1	51	2.793	146.1

**Table 2:** Related work on multihop LoRaWAN.

Paper	Lora/LoRaWAN	T	S	P	Scenario
Abrardo and Pozzebon [21]	LoRa			x	underground
Anedda et al. [20]	LoRaWAN		x		general
Aslam et al. [37]	LoRaWAN			x	smart cities
Azhari [52]	LoRa			x	linear
Bezunartea et al. [53]	LoRa			x	general
Bor et al. [17]	LoRa			x	University Campus
Choi et al. [36]	LoRaWAN			x	general
Dias and Grilo [24]	LoRaWAN			x	linear and bootleneck
Diop and Pham [33]	LoRa			x	farm
Duong and Kim [22]	LoRaWAN			x	University Campus
Dwijaksara et al. [30]	LoRaWAN		x		general
Ebi et al. [27]	LoRa / LoRaWAN			x	urban underground monitoring
Farooq [40]	LoRa		x		general
Flauzac [54]	Lora/LoRaWAN			x	general
Haubro et al. [29]	LoRa			x	general
Huh and Kim [55]	LoRaWAN			x	urban/industrial
Kim and Jang [56]	LoRaWAN			x	general
Lee and Ke [23]	LoRa			x	University Campus
Liao et al. [18]	LoRa	x	x	x	multiple-building area networks (MBAN)
Lundell et al. [19]	LoRaWAN			x	linear and warehouse
Mai and Kim [25]	LoRaWAN			x	smart cities
Misbahuddin et al. [39]	LoRaWAN	x	x		ring
Ochoa et al. [38]	LoRa	x			general
Sartori et al. [28]	LoRa			x	general
Sisinni et al. [31]	LoRaWAN			x	industrial
Sisinni et al. [32]	LoRaWAN			x	industrial
Tanjung et al. [35]	LoRaWAN		x		general
Weiwei Zhou and Wang [34]	LoRaWAN			x	urban
Zhu et al. [26]	LoRa		x	x	general

**Table 3:** Devices classification.

Device	Radio	Energy	Smart	Details
End-device	- Single-Channel	- Constrained - Non-Constrained	- Non-Smart	- Create new LoRaWAN packet
Relay-Device	- Single-Channel - Multi-Channel	- Constrained - Non-Constrained	- Smart - Non-Smart	- Forward packets - Create new LoRaWAN packet
Router-Device	- Single-Channel - Multi-Channel	- Constrained - Non-Constrained	- Smart	- Implement a routing protocol - Create new LoRaWAN packet
Main Gateway	- Multi-Channel	- Non-Constrained	- Smart - Non-Smart	- Connected to the Internet - Split networks
Relay-Gateway	- Multi-Channel	- Non-Constrained	- Smart - Non-Smart	- Forward packets - Not connected to the Internet
Router-Gateway	- Multi-Channel	- Non-Constrained	- Smart	- Implement a routing protocol - Not connected to the Internet

**Table 4:** Intermediate device functions and characteristics.

Paper	Device	Radio	Energy Constrained	Smart
Abrardo and Pozzebon [21]	Router-Device	Single	Yes	Yes
Anedda et al. [20]	Router-Device	Single	Yes	Yes
Aslam et al. [37]	Relay-Device	Single	No	No
	Relay-Gateway			
Bor et al. [17]	Router-Device	Single	Yes	Yes
Choi et al. [36]	Relay-Device	Single	Yes	Yes
Dias and Grilo [24]	Router-Device	Single	No	Yes
Diop and Pham [33]	Relay-Device	Single	Yes	Yes
Duong and Kim [22]	Relay-Device	Single	Yes	Yes
Dwijaksara et al. [30]	Router-Gateway	Multi	No	Yes
Ebi et al. [27]	Router-Device	Single	Yes	No
	Relay-Gateway	Multi		
Haubro et al. [29]	Router-Device	Single	Yes	Yes
Lee and Ke [23]	Router-Device	Single	No	Yes
Liao et al. [18]	Router-Device	Single	Yes	Yes
Lundell et al. [19]	Router-Device	Single	Yes	Yes
Mai and Kim [25]	Router-Device	Single	Yes	Yes
Sartori et al. [28]	Router-Device	Single	Yes	Yes
Sisinni et al. [31]	Relay-Gateway	Multi	No	No
Sisinni et al. [32]	Relay-Device	Multi	No	Yes
Tanjung et al. [35]	Relay-Device	Single	Yes	Yes
Weiwei Zhou and Wang [34]	Relay-Device	Single	Yes	Yes
Zhu et al. [26]	Router-Device	Single	Yes	Yes

**Table 5:** Topology summary.

Architecture	Scenario	Scenario Details	Related Work
Relay-Device (Figure 6a)	- Smart Building - Industrial	- Improve connection - Access to power supply	Aslam et al. [37] Choi et al. [36] Diop and Pham [33] Duong and Kim [22] Sisinni et al. [32] Tanjung et al. [35] Weiwei Zhou and Wang [34]
Relay-Gateway (Figure 6b)	- Smart Farm	- Connect remote areas - Concentrate data traffic	Aslam et al. [37] Sisinni et al. [31]
Relay-Device Relay-Gateway (Figure 6c)	- Smart City	- Improve coverage area - Concentrate data traffic - Battery powered devices	None
Router-Device (Figure 7a)	- Smart Building - Industrial	- Improve connection - Access to power supply - Multiple barriers scenario	Abrardo and Pozzebon [21] Anedda et al. [20] Bor et al. [17] Dias and Grilo [24] Haubro et al. [29] Lee and Ke [23] Liao et al. [18] Lundell et al. [19] Mai and Kim [25] Sartori et al. [28] Zhu et al. [26]
Router-Gateway (Figure 7b)	- Smart Farm - Smart City	- Connect remote areas - Concentrate data traffic	Dwijaksara et al. [30]
Router-Device Router-Gateway (Figure 7c)	- Underground	- Improve coverage area - Concentrate data traffic	None
Hybrid	- Smart Farm - Underground	- Improve connection - Improve coverage area - Concentrate data traffic	Ebi et al. [27]
